# Tectorigenin inhibits RANKL‐induced osteoclastogenesis via suppression of NF‐κB signalling and decreases bone loss in ovariectomized C57BL/6

**DOI:** 10.1111/jcmm.13801

**Published:** 2018-07-31

**Authors:** Chiyuan Ma, Kai Xu, Jiahong Meng, Jisheng Ran, Safwat Adel Abdo Moqbel, An Liu, Shigui Yan, Lidong Wu

**Affiliations:** ^1^ Department of Orthopaedic Surgery Second Affiliated Hospital Zhejiang University School of Medicine Hangzhou China

**Keywords:** NF‐κB, osteoclast, osteoporosis, tectorigenin

## Abstract

Metabolism of bone is regulated by the balance between osteoblast‐mediated bone formation and osteoclast‐mediated bone resorption. Activation of osteoclasts could lead to osteoporosis. Thus, inhibiting the activity of osteoclasts becomes an available strategy for the treatment of osteoporosis. Tectorigenin is an extract of Belamcanda chinensis In the present study, the anti‐osteoclastogenesis effects of tectorigenin were investigated in vitro and in vivo. The results showed preventive and therapeutic effects of tectorigenin at concentrations of 0, 10, 40, and 80 μmol/L in the maturation and activation of osteoclasts. A signalling study also indicated that tectorigenin treatment reduces activation of NF‐κB signalling in osteoclastogenesis. Animal experiment demonstrated that tectorigenin treatment (1‐10 mg/kg, abdominal injection every 3 days) significantly inhibits bone loss in ovariectomized C57BL/6. Our data suggest that tectorigenin is a potential pharmacological choice for osteoporosis.

## 1 INTRODUCTION

Osteoporosis has been one of the most common health problems around world.^1^ Fractures associated with poor bone quality has been a large burden to health systems, especially in ageing populations and postmenopausal female populations.^2^ Metabolism of bone is regulated by the balance between osteoblast‐mediated bone formation and osteoclast‐mediated bone resorption.^3^, ^4^ Oestrogen loss in postmenopausal women promotes activation of osteoclasts.^5^ Osteoclast activation could result in enhanced bone resorption, leading to imbalance of bone metabolism and resulting in bone diseases. Differentiated from hematopoietic precursor cells of monocyte‐macrophage lineage, osteoclasts are the sole bone‐resorbing multinucleated giant cells.^6^ There are several targets within osteoclast for intervention to prevent bone loss. Biophosphonates, as one of the most widely used anti‐osteoporosis drugs, inhibit attachment to bone matrix and induce osteoclast apoptosis.^7^, ^8^ However, serious gastrointestinal reactions from this kind of drug remain unpleasant for patients.^9^

The receptor activator of nuclear factor‐κB (NF‐κB) ligand (RANKL) and macrophage colony‐stimulating factor (M‐CSF) promote osteoclast differentiation and functioning.^6^, ^10^, ^11^ Increased RANKL levels could activate multiple downstream pathways, including NF‐κB, MAPKs, followed by the activation of transcription factors including AP‐1 and NFATc1, thereby enhancing the differentiation and activation of monocyte‐macrophage precursors into osteoclasts.^12^

Tectorigenin (Chemical Abstracts Service number 548‐77‐6, C16H12O6, molecular weight, 300.26) is an extract of Belamcanda chinensis of the iris species. Its potential usage in anti‐inflammatory and antioxidant activity was reported before,^13^, ^14^ and more specifically, its anti‐inflammatory effect in osteoarthritis and NF‐κB signalling blockage.^15^ In this study, the effect of tectorigenin on RANKL‐induced NF‐κB activation and osteoclastogenesis was investigated in vitro, plus its anti‐osteoporosis action in an ovariectomized (OVX) mice model.

## 2 METHODS AND MATERIALS

### 2.1 Reagents and materials

Tectorigenin (purity >98% by HPLC) was obtained from Tauto Biotech (Shanghai, China). Recombinant mouse RANKL and M‐CSF were purchased from R&D Systems (Minneapolis, MN, USA). Alpha Modification of Eagle's Medium (α‐MEM), foetal bovine serum (FBS), penicillin, and streptomycin were purchased from Gibco (Rockville, MD, USA). Cell Counting Kit‐8 (CCK‐8) was purchased from Dojindo Molecular Technology (Japan). Tartrate‐resistant acid phosphatase (TRAP) staining kit, dimethyl sulphoxide (DMSO), ethylenediaminetetraacetic acid (EDTA) were obtained from Sigma‐Aldrich (St Louis, MO, USA). NF‐κB p65 (CST 8242), phosphor‐NF‐κB p65 (CST 3033), IκBα (CST 4812), and phosphor‐IκBα (CST 2859) antibodies were purchased from Cell Signalling Technology (Beverly, MA, USA). β‐actin (SC‐47778) antibodies was purchased from Santa Cruz Biotechnology.

### 2.2 Animals

C57BL/6 mice (6‐week‐old, female, Animal Center of Zhejiang University) were used in the present study. Water and food were provided in the facility. All the experiments that involved animals were approved by the Ethics Committee of the Second Affiliated Hospital, Zhejiang University School of Medicine, Hangzhou, China (2015‐107) and conducted in accordance with the Declaration of Helsinki.

### 2.3 Cell Counting Kit (CCK‐8) assay

CCK‐8 was used to evaluate the cytotoxicity of tectorigenin according to the manufacturer's instruction. Briefly, bone marrow mononuclear (BMM) cells or RAW264.7 cells were seeded in 96‐well plates at a density of 3000/well. After incubation with concentrations of 0, 10, 40, 80, or 160 μmol/L of tectorigenin for 24 hour or 48 hour, cells were incubated with 10 μL CCK‐8 for 4 hour. The optical density (OD) was read at a wavelength of 450 nm.

### 2.4 BMM cells isolation and cell treatment

Bone marrow mononuclear cells were prepared as other previous studies.^16^, ^17^ In brief, 6‐week‐old C57/BL6 mice were sacrificed. Cells extracts from femurs and tibias of one mouse were incubated in cell culture (α‐MEM containing 10% FBS, 100 U/mL penicillin, and 100 mg/mL streptomycin) with 30 ng/mL M‐CSF in one T‐75 cm^2^ flask. The medium was changed every 2 or 3 days. When the cells reached 90% confluence, cells were washed with PBS and trypsinized at 37°C with 5% CO_2_ for 30 minutes for harvest. The harvested cells were classified as BMM cells.

Cells were seeded in plates for analysis. Subconfluent cells were pretreated with tectorigenin at concentrations of 0, 10, 40, or 80 μmol/L for 1 hour then incubated with or without RANKL (25, 50, or 100 ng/mL) according to the study design.

All medium used for BMM cells in this study contained 30 ng/mL M‐CSF, while medium for RAW264.7 contained no M‐CSF.

### 2.5 Osteoclastogenesis analysis

Bone marrow mononuclear cells were seeded in 24‐well plates (5 × 10^5^ cells/well) and treated with 0, 10, 40, or 80 μmol/L of tectorigenin in the presence of RANKL (50 ng/mL) for 4 days. RAW264.7 cells were also treated with tectorigenin (0, 10, 40, 80 μmol/L) in the presence of RANKL (50 ng/mL) for 4 days. Cells were then fixed and stained for TRAP assay according to the manufacturer's protocol.

### 2.6 Bone resorption pit assay

Bone marrow mononuclear cells (1 × 10^4^ cells/well) were seeded in hydroxyapatite‐coated plates (3989, Corning, St. Lowell, MA, USA). Subconfluent cells were incubated with tectorigenin at concentrations of 0, 10, 40, or 80 μmol/L in the presence of RANKL (50 ng/mL) for 7 days. The medium was changed every 3 days. After the treatment, the plates were washed with 10% bleach solution and air‐dried at RT. Osteoclast resorption area was observed using a light microscope.

### 2.7 RNA extraction and real‐time PCR

Bone marrow mononuclear cells or RAW264.7 cells were seeded in 12‐well plates at a density of 1 × 10^6^ cells/well and treated with tectorigenin at concentrations of 0, 10, 40, or 80 μmol/L in the presence or absence of RANKL (50 ng/mL) for 48 hour. We used TRIzol reagent (Invitrogen, Carlsbad, CA, USA) to extract RNA from cells according to the protocol. Total RNA was used to synthesize cDNA by reverse transcription (cDNA Synthesis Kit, Takara Biotechnology, Otsu, Japan). Power SYBR Green PCR Master Mix (Takara Biotechnology) was used for real‐time PCR. The expression of TRAP, NFATc1, Cts K, and MMP9 was analysed using primer sequences listed in Table 1. GAPDH worked as endogenous controls. For real‐time PCR, 10 μL reaction mixture contained SYBR Green and each primer. PCR was set using StepOnePlus system (Applied Biosystems). The programme included one cycle of denaturation at 95°C for 1 minutes and 40 cycles of denaturation at 95°C for 15 seconds, primer annealing, and extension at 63°C for 25 seconds, followed by melt curve analysis. Data were analysed for fold difference using formula: 2^−▵▵CT^.

**Table 1:** Primers used for real‐time PCR

Gene	Forward	Reverse
Mouse TRAP	CTGGAGTGCACGATGCCAGCGACA	TCCGTGCTCGGCGATGGACCAGA
Mouse NFATc1	CCGTTGCTTCCAGAAAATAACA	TGTGGGATGTGAACTCGGAA
Mouse Cts K	CTTCCAATACGTGCAGCAGA	TCTTCAGGGCTTTCTCGTTC
Mouse MMP9	AGTTTGGTGTCGCGGAGCAC	TACATGAGCGCTTCCGGCAC
Mouse GAPDH	GCAAGTTCAACGGCACAG	CGCCAGTAGACTCCACGAC

TRAP, tartrate‐resistant acid phosphatase.

### 2.8 Protein extraction and Western blot analysis

Bone marrow mononuclear cells were used for Western blot analysis. Cells were pretreated with 0, 10, 40, or 80 μmol/L of tectorigenin for 1 hour, and then stimulated with 50 ng/mL RANKL for 1 hour for detection of NF‐κB p65, phosphor‐NF‐κB p65, IκBα, and phosphor‐IκBα. We used radioimmunoprecipitation assay (RIPA) containing protease and phosphatase inhibitors to prepare cell extract. Equal amounts of cell extract were separated by 10% SDS‐PAGE, and electro‐transferred to polyvinylidene difluoride membranes. After blocking with 5% bull serum albumin (BSA, Sigma‐Aldrich, St, Louis, MO, USA) for 2 hours, the membranes were blotted with primary antibodies at 4°C overnight, then incubated for 1 hour with secondary antibody. After that, signals were detected using West Dura Extended Duration Substrate with exposure to X‐ray film.

### 2.9 Luciferase reporter gene analysis

RAW264.7 cells were used in this experiment. Cells were first stably transfected with an NF‐κB luciferase reporter construct (Promega, Madison, WI) firstly. Then, transfected cells were pretreated with tectorigenin (0, 10, 40, or 80 μmol/L) for 1 hour and then stimulated with RANKL (50 ng/mL) for 6 hour. After that, the luciferase assay system (Promega) was used to measure the luciferase activity.

### 2.10 Immunofluorescence microscopy

Coexpression of NF‐κB p65 was carried out using fluorochrome‐conjugated antibodies. RAW264.7 cells cultured 24‐well plate (5000/well) were fixed in 4% paraformaldehyde for 10 minutes, and permeabilized for 5 minutes with 0.1% v/v Triton X‐100. Cells were incubated with primary antibody (sc‐8008) at 4°C overnight, washed, and then incubated with fluorochrome‐conjugated secondary antibody for 2 hour in the dark. Coverslips were mounted onto glass slides using DAPI‐containing mounting medium.

### 2.11 Induction of osteoporosis in mice

A mice model of osteoporosis was developed by bilateral ovariectomies in 6‐week‐old female C57BL/6 as previously described in other studies.^18^, ^19^ One week after surgery, all the mice were divided into four groups with 10 mice per group: the mice that underwent sham surgery were grouped as Sham; mice with bilateral ovariectomies were divided randomly into three groups: OVX, OVX + Tectorigenin 1 mg/kg, and OVX + Tectorigenin 10 mg/kg. Mice in OVX and Sham groups were treated with vehicle. Mice in OVX + Tectorigenin 1 mg/kg received low dose of tectorigenin (1 mg/kg), while OVX + Tectorigenin 10 mg/kg was treated by high dose of tectorigenin (10 mg/kg). All the tectorigenin or vehicle was delivered by abdominal injection every 3 days. All mice were sacrificed by excess amount of chloral hydrate after 6‐week treatment. The distal femurs were preserved and fixed in 4% paraformaldehyde solution. The timeline of animal experiments is shown in Figure 5.

### 2.12 Micro‐computed tomography scanning

A high‐resolution micro‐CT scanner (vivaCT80, Scanco Medical, Zurich, Switzerland) was used to scan the fixed distal femur with a source voltage of 50 kVp, electric current of 500 μA, rotation step of 0.7°. MicView software was used to reconstruct the images. The region between articulating surface of condyles and growth plate was analysed. Bone mineral density (BMD), bone volume fraction (BV/TV), trabecular number (Tb.N.), and trabecular separation (Tb.Sp.) were measured by CT Analyzer software.

### 2.13 Histological analysis

Samples fixed with 4% paraformaldehyde solution were decalcified and embedded in paraffin, sectioned at 5 μm thickness. The sections were stained with haematoxylin and eosin (H&E) and TRAP.

### 2.14 Statistical analysis

The results are presented as mean ± standard deviation of three or more experiments. Statistical differences were performed with SPSS 12.0 version. One‐way ANOVA with a subsequent post hoc Tukey's test was used for multiple comparisons. *P* < 0.05 is considered to be significant with statistical meaning.

## 3 RESULTS

### 3.1 Cytotoxicity of tectorigenin and preventive effect on RANKL‐induced osteoclast differentiation and bone resorption in vitro

**Figure 1:**
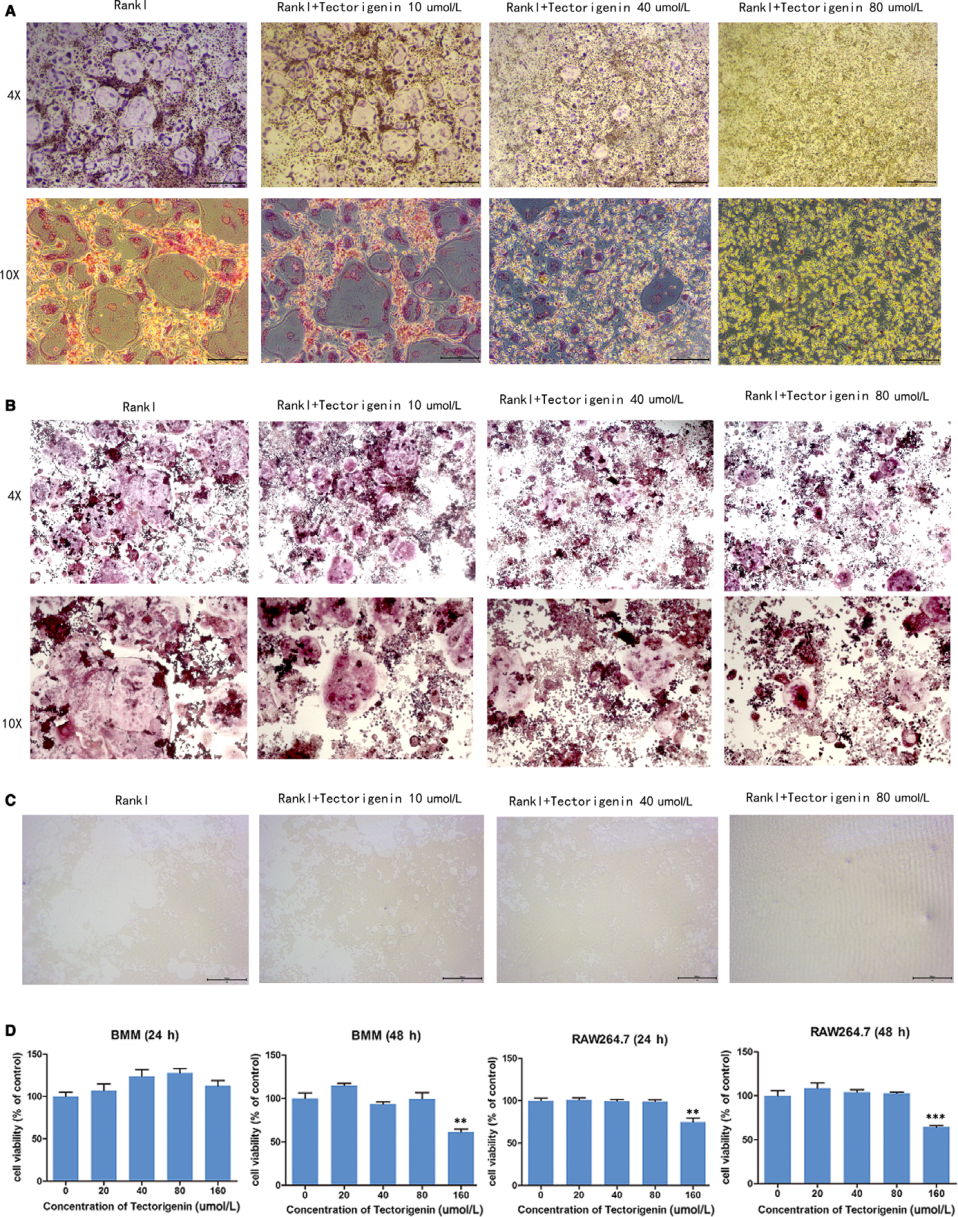
Cytotoxicity of tectorigenin and preventive effect on RANKL‐induced osteoclast differentiation. A, bone marrow mononuclear (BMM) cells were treated with 0, 10, 40, or 80 μmol/L of tectorigenin in the presence of RANKL (50 ng/mL) and M‐CSF (30 ng/mL) for 4 d. B, RAW264.7 cells were treated with similar concentrations of tectorigenin in the presence of RANKL (50 ng/mL) for 4 d. We assessed the osteoclast differentiation by tartrate‐resistant acid phosphatase (TRAP) stain. C, BMM cells were incubated with tectorigenin at similar concentrations in the presence of RANKL (50 ng/mL) for 7 d to observe the resorption area on hydroxyapatite‐coated surfaces. D, Cytotoxicity of tectorigenin was assessed by CCK‐8 assay. Significance was calculated by a one‐way ANOVA with a post hoc Tukey's multiple comparisons test. **P < 0.01, ***P < 0.001 vs 0 μmol/L concentration tectorigenin treated group

To evaluate cytotoxicity of tectorigenin, RAW264.7 cells and BMM cells were treated with tectorigenin at concentrations of 0, 10, 40, 80, or 160 μmol/L for 24 hour or 48 hour. CCK‐8, which was used to measure the cell viability, revealed that tectorigenin caused no significant cytotoxicity on BMM cells or RAW267.4 cells at concentrations lower than 80 μmol/L (Figure 1D). Therefore, 80 μmol/L was used as the highest concentration in the following experiments.

To measure the preventive effect of tectorigenin on RANKL‐induced osteoclast differentiation, BMM cells and RAW264.7 cells were treated with 0, 10, 40, or 80 μmol/L of tectorigenin in the presence of RANKL (50 ng/mL). After 4‐day incubation, the osteoclast differentiation of BMM cells (Figure 1A) and RAW264.7 cells (Figure 1B) by TRAP stain. Results showed that treatment of tectorigenin reduced osteoclast differentiation in both cells with a tectorigenin‐dose‐dependent manner. Afterwards, we assessed whether tectorigenin could inhibit function of osteoclast. BMM cells (1 × 10^4^ cells/well) were seeded in hydroxyapatite‐coated plates. Then, subconfluent cells were incubated with tectorigenin at concentrations of 0, 10, 40, or 80 μmol/L in the presence of 50 ng/mL RANKL for 7 days. As shown in Figure 1C, tectorigenin significantly reduced resorption area stimulated by RANKL.

### 3.2 Effect of tectorigenin on RANKL‐stimulated osteoclast‐specific gene expression

**Figure 2:**
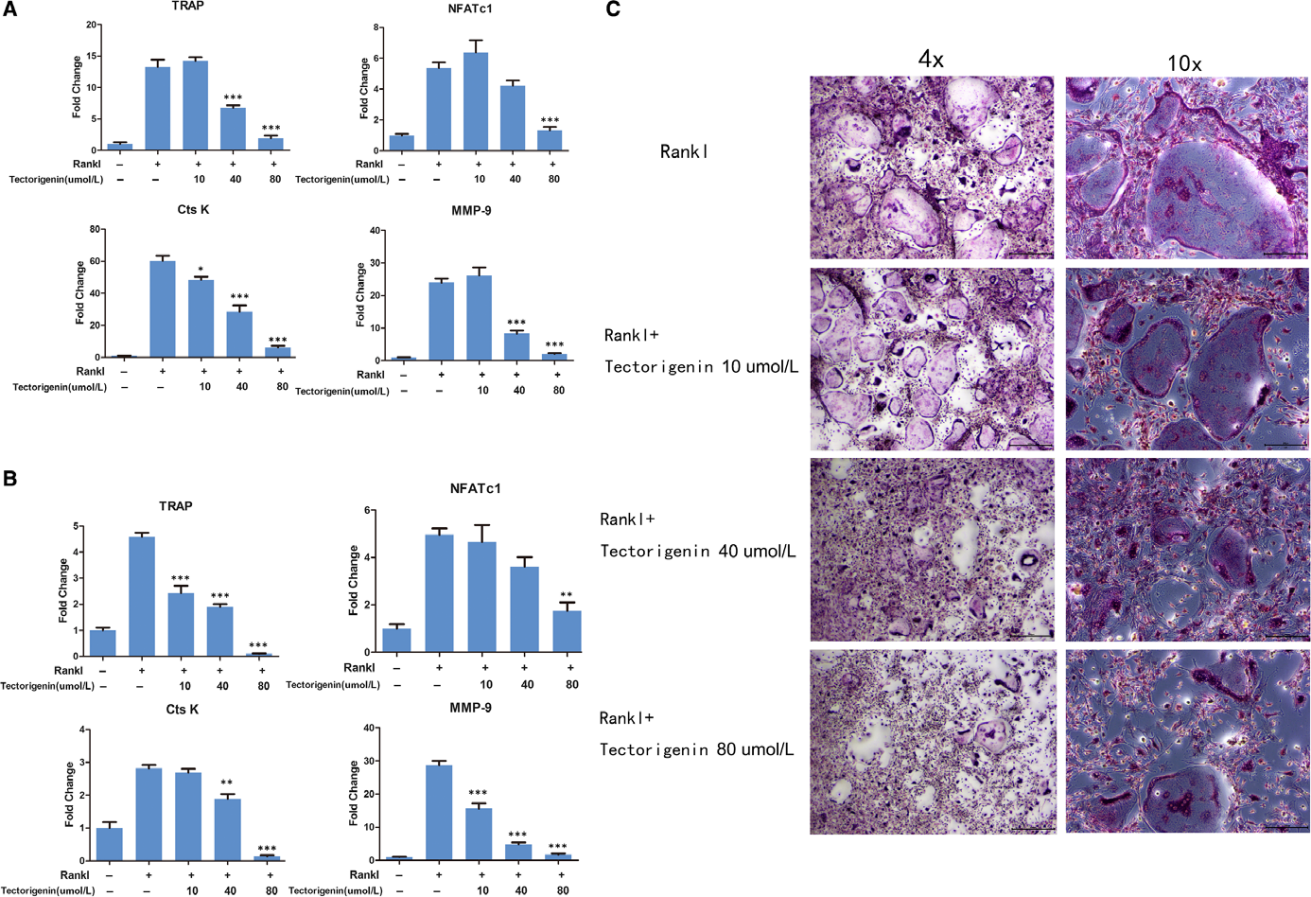
Effect of tectorigenin on RANKL‐stimulated osteoclast‐specific gene expression and therapeutic effect of tectorigenin in vitro. A, bone marrow mononuclear (BMM) cells were treated with tectorigenin at concentrations of 0, 10, 40, or 80 μmol/L in the presence of RANKL (50 ng/mL) and M‐CSF (30 ng/mL) for 48 h. B, RAW264.7 cells were treated with similar concentrations of tectorigenin and RANKL (50 ng/mL) for 48 h. Expression of osteoclast‐specific genes including tartrate‐resistant acid phosphatase (TRAP), NFATc1, cathepsin K (Cts K), and MMP9 were investigated by Real‐time PCR. Significance was calculated by a one‐way ANOVA with a post hoc Tukey's multiple comparisons test. *P < 0.05, **P < 0.01, ***P < 0.001 vs RANKL+ 0 μmol/L concentration tectorigenin treated group. C, First, BMM cells were stimulated by RANKL (50 ng/mL) in the presence of M‐CSF (30 ng/mL) for 4 d until osteoclasts were observed. Cells were then incubated with tectorigenin at similar concentrations at present of RANKL (50 ng/mL) for another 4 d for TRAP staining

To investigate the effect of tectorigenin on RANKL‐stimulated osteoclast‐specific gene expression, BMM cells and RAW264.7 were pretreated with tectorigenin at concentrations of 0, 10, 40, or 80 μmol/L for 1 hour, and then stimulated by RANKL (50 ng/mL) for 48 hour. Up‐regulation of osteoclast‐specific genes, including TRAP, NFATc1, cathepsin K (Cts K), and MMP9, were investigated by Real‐time PCR. As shown in Figure 2, expression of TRAP, NFATc1, Cts K, and MMP9 was down‐regulated by tectorigenin treatment. Combined data in Figure 1, the data demonstrated that tectorigenin exhibited a preventive effect on osteoclastogenesis.

### 3.3 Therapeutic effect of tectorigenin on RANKL‐induced osteoclast differentiation in vitro

To determine whether tectorigenin could also help in treating RANKL‐induced osteoclast differentiation, BMM cells were stimulated by RANKL (50 ng/mL) for the first 4 days until osteoclasts were observed. Cells were then incubated with tectorigenin at concentrations of 0, 10, 40, or 80 μmol/L with RANKL (50 ng/mL) for another 4 days. Tartrate‐resistant acid phosphatase stain showed that tectorigenin reduced osteoclast differentiation induced by RANKL stimulation (Figure 2B). Our results demonstrated that tectorigenin showed a therapeutic role when the differentiation of osteoclasts was already initiated by RANKL.

### 3.4 Effect of tectorigenin on RANKL‐induced activation of NF‐κB signalling in vitro

**Figure 3:**
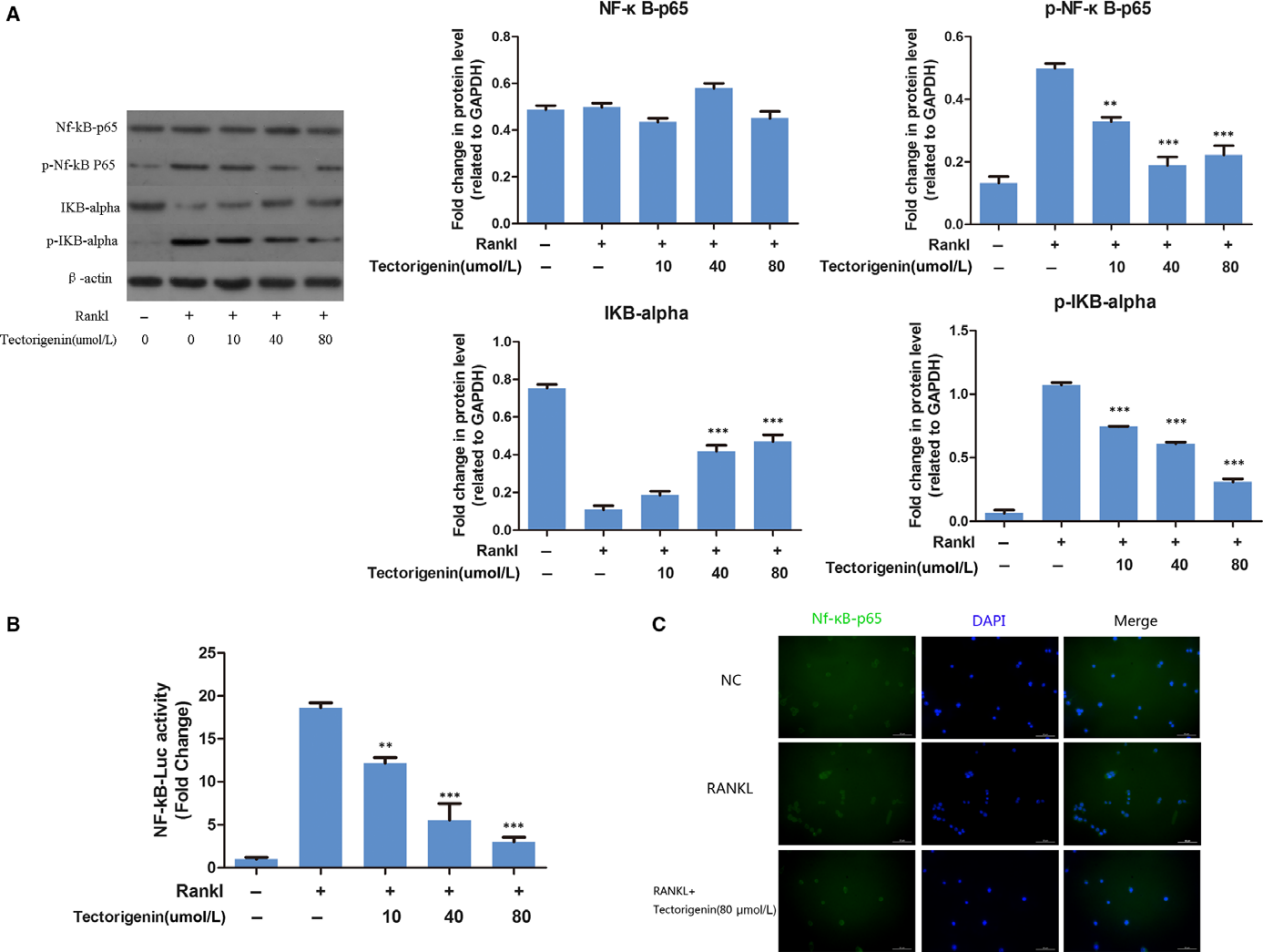
Effect of tectorigenin on RANKL‐induced activation of NF‐κb signalling. A, bone marrow mononuclear (BMM) cells were pretreated with tectorigenin at concentration of 0, 10, 40, or 80 μmol/L for 1 h, and then stimulated with 50 ng/mL RANKL for 1 h. Phosphorylation and protein level of NF‐κB p65 and IκBα were measured by Western blot. B, RAW264.7 cells were pretreated with similar concentration of tectorigenin for 1 h, and then stimulated with 50 ng/mL RANKL for 6 h for luciferase reporter gene assay. C, RAW264.7 cells were pretreated with tectorigenin (80 μmol/L) for 2 h, and then stimulated with 50 ng/mL RANKL for 30 min for immunofluorescence microscopic analysis. Significance was calculated by a one‐way ANOVA with a post hoc Tukey's multiple comparisons test. **P < 0.01, ***P < 0.001 vs RANKL+ 0 μmol/L concentration tectorigenin treated group

Considering the importance of NF‐κB signalling in osteoclast differentiation,^20^ we investigated the effect of tectorigenin on RANKL‐induce activation of NF‐κB signalling on BMM cells in vitro. In Western blot assay, phosphorylation and protein levels of NF‐κB p65 and IκBα were measured in the present study. BMM cells were pretreated with tectorigenin at concentration of 0, 10, 40, or 80 μmol/L for 1 hour, and then stimulated with 50 ng/mL RANKL for 1 hour. As shown in Figure 3A, tectorigenin treatment significantly reduced phosphorylation level of NF‐κB p65 and IκBα and degradation of IκBα. To confirm the Western blot data, the effect of tectorigenin on transcriptional activity of NF‐κB induced by RANKL was examined using a luciferase reporter gene assay. RAW264.7 cells were pretreated with 0, 10, 40, 80 μmol/L tectorigenin for 1 hour, and then stimulated with 50 ng/mL RANKL for 6 hour for analysis. The luciferase reporter gene assay had a similar result (Figure 3B). The increased transcriptional activity of NF‐κB induced by RANKL was greatly decreased by tectorigenin treatment with a dose‐dependent manner. Furthermore, the location and concentration of NF‐κB p65 was visualized using immunofluorescence microscopic analysis, and this kind of inhibition was also observed (Figure 3C). These results indicate that tectorigenin inhibits RANKL‐induced activation of NF‐κB signalling in vitro.

### 3.5 Effect of tectorigenin on OVX‐induced bone loss in vivo

**Figure 4:**
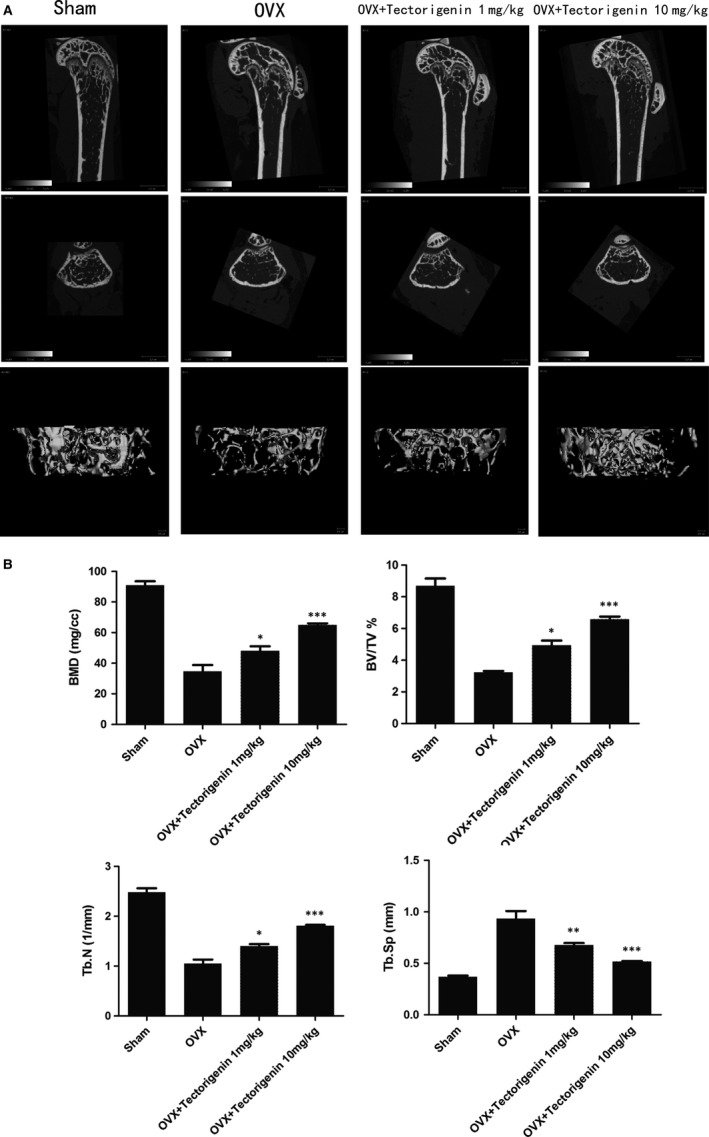
Effect of tectorigenin on ovariectomized (OVX)‐induced bone loss in vivo. Mice in OVX and Sham groups were treated with vehicle. Mice in OVX + Tectorigenin 1 mg/kg received low dose of tectorigenin (1 mg/kg), while OVX + Tectorigenin 10 mg/kg was treated by high dose of tectorigenin (10 mg/kg). All tectorigenin or vehicle was delivered by abdominal injection every 3 d. The distal femurs were subjected to Micro‐CT to evaluate the bone loss level. Bone mineral density (BMD), bone volume fraction (BV/TV), trabecular number (Tb.N.), and trabecular separation (Tb.Sp.) were measured by CT Analyzer software. Significance was calculated by a one‐way ANOVA with a post hoc Tukey's multiple comparisons test. *P < 0.05, **P < 0.01, ***P < 0.001 vs OVX group

To investigate effects of tectorigenin on OVX‐induced bone loss in vivo, we developed mice model of osteoporosis by bilateral ovariectomies in 6‐week‐old female C57BL/6. Mice in OVX and Sham groups were treated with vehicle. Mice in OVX + Tectorigenin 1 mg/kg received low dose of tectorigenin (1 mg/kg), while OVX + Tectorigenin 10 mg/kg was treated by high dose of tectorigenin (10 mg/kg). All the tectorigenin or vehicle was delivered by abdominal injection every 3 days. All mice were sacrificed by excess amount of chloral hydrate after 6‐week treatment. The distal femurs were preserved and fixed in 4% paraformaldehyde solution. Micro‐CT images showed the bone loss of trabecular bone was reduced by tectorigenin treatment in OVX mice (Figure 4A). Image analysis indicated that decrease of BMD, BV/TV, Tb.N and increase of Tb.Sp induced by OVX were all reduced by tectorigenin treatment (Figure 4B).

**Figure 5:**
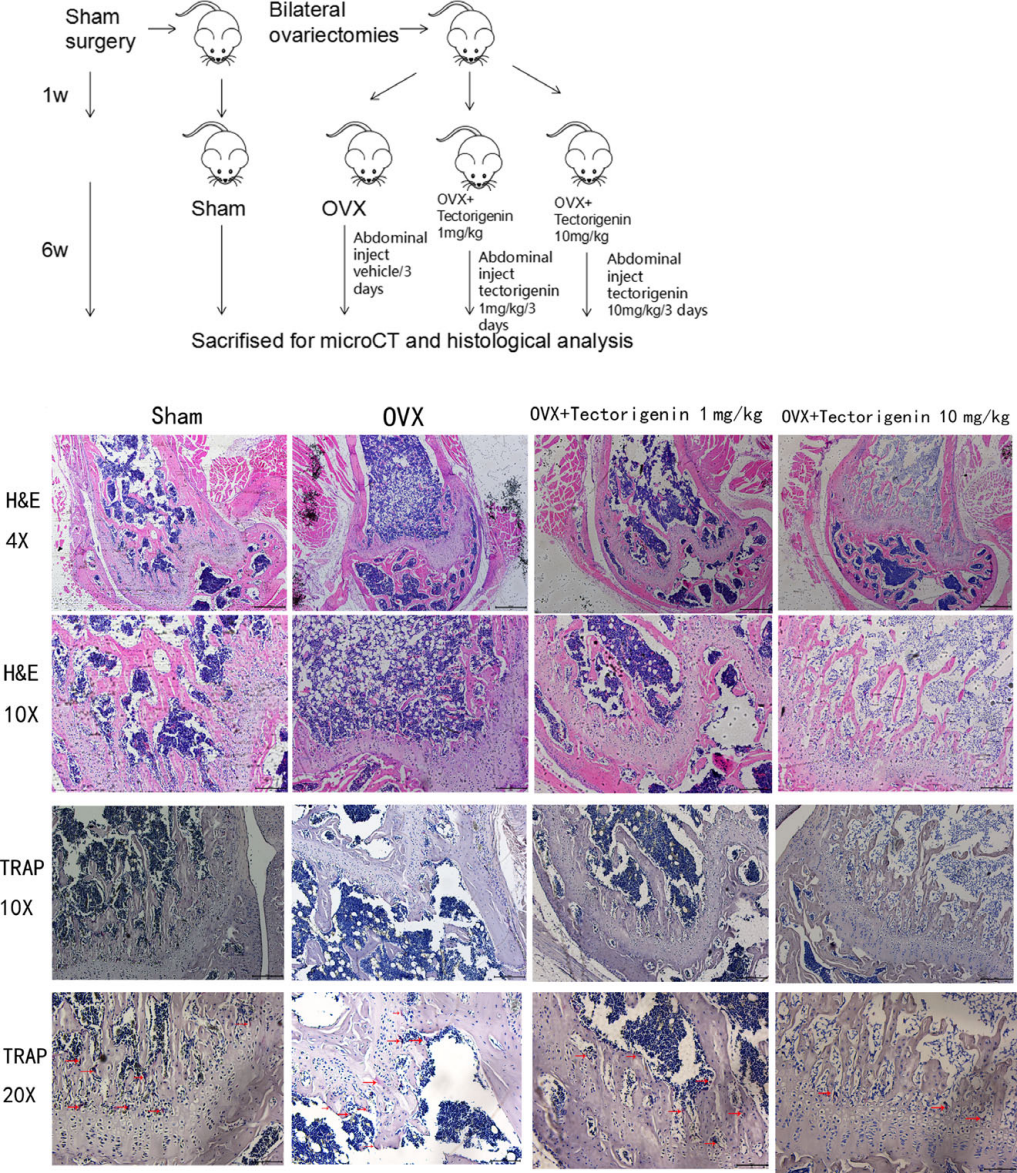
Experimental timeline and histological analysis of in vivo effect of tectorigenin. The distal femurs were decalcified and embedded in paraffin, sectioned at 5 μm thickness. The sections were stained with haematoxylin and eosin (H&E) and tartrate‐resistant acid phosphatase (TRAP). The red arrows in TRAP stained sections illustrates the red‐stained osteoclasts

Histological assessment confirmed the results from Micro‐CT. H&E staining (Figure 5) showed significant reduced trabecular numbers in the OVX mice compared with the Sham group. Improvements were observed in the OVX + Tectorigenin 10 mg/kg group. Furthermore, TRAP staining also indicated decreased number of osteoclasts between trabeculars (Figure 5). Collectively, these data indicated that tectorigenin reduced OVX‐induced bone loss in vivo.

## 4 DISCUSSION

Balance between osteoblast‐mediated bone formation and osteoclast‐mediated bone resorption induces bone turnover and remodelling.^4^ Thus, inhibiting the activity of osteoclasts becomes an available strategy for the treatment of osteoporosis. We report that tectorigenin exerts anti‐osteoporosis in an OVX mice model and inhibits RANKL‐induced osteoclastogenesis through inhibition of NF‐κB signalling in vitro.

We first evaluated the effect of tectorigenin on osteoclast differentiation on BMM cells and RAW264.7 cells. Tartrate‐resistant acid phosphatase staining showed that tectorigenin treatment had an obvious protective effect on RANKL‐induced osteoclast differentiation in vitro at concentrations lower than cytotoxic levels. TRAP‐positive and multinucleate cell number was decreased along with the increased concentration of tectorigenin, and nearly no mature osteoclasts were visualized in high concentration (80 μmol/L) treated group (Figure 1A). Meanwhile, number of osteoclast was increased along with increased concentration of RANKL at same concentration of tectorigenin (Figure S1), which means tectorigenin effect on osteoclastogenesis is also RANKL‐concentration‐dependent. Real‐time PCR was then performed to measure the change of osteoclast marker gene expression (TRAP, NFATc1, Cts K, and MMP9) in the treatment. To avoid potential bias, we also performed this experiment on BMM cells and RAW264.7 cells. Results showed that tectorigenin inhibited RANKL‐stimulated osteoclast‐specific gene expression at non‐cytotoxic concentrations.

However, when reviewing the above experiments, we realized that all the cells were incubated with RANKL in the presence of tectorigenin. Pharmacologic intervention is hardly performed before diagnosis of osteoporosis in clinical practice. Therefore, to simulate the pharmacologic intervention after diagnosis of osteoporosis in clinical practice, we added tectorigenin into the medium after the appearance of osteoclasts to evaluate its therapeutic effect. Tartrate‐resistant acid phosphatase staining showed less osteoclasts in tectorigenin‐treated groups, which indicated that tectorigenin treatment had a therapeutic effect on RANKL‐induced osteoclast differentiation in vitro.

The reduction of oestrogen levels is related to the development of postmenopausal osteoporosis in female populations.^5^, ^21^ This study simulates menopause by removing the ovaries in female C57BL/6 mice, resulting in postmenopausal osteoporosis. Micro‐CT and histological analysis indicated that 6‐week tectorigenin treatment had an obvious protective effect on OVX‐induced bone loss. Histological analysis also showed less osteoclasts in the tectorigenin treated mice.

NF‐κB signalling is one of the major pathways involved in osteoclastogenesis.^6^ When the signalling is activated by RANKL, phosphorylation of NF‐κB p65 and IκBα and degradation of IκBα contribute to the activation and nuclear translocation of NF‐κB p65. NF‐κB p65 could enhance osteoclast‐specific gene expression, thus resulting in osteoclast differentiation and maturation.^17^, ^22^ A study by Rong Zeng et al^23^ indicated that alternative NF‐κB signalling also plays a role in controlling the independent processes of osteoclast mitochondrial biogenesis. NF‐κB signalling is involved in immune responses and function regulation of osteoclasts as well.^24^ NF‐κB signalling has already been one important target for osteoporotic pharmacological intervention.^22^, ^25^ It has been reported in other studies that tectorigenin inhibit activation of NF‐κB on other cells.^15^, ^26^, ^27^ In this study, we evaluated the activation of NF‐κB signalling in the tectorigenin treatment on BMM cells. Our data demonstrated that tectorigenin could inhibit degradation of IκBα and phosphorylation of NF‐κB p65 and IκBα induced by RANKL stimulation at non‐cytotoxicity concentrations. Furthermore, transcription analysis revealed a similar result. These suggested tectorigenin could suppress RANKL‐stimulated osteoclastogenesis via regulation of NF‐κB activation.

The traditional pharmacologic therapy consists of drugs like teriparatide or bisphosphonate, of which long‐term safety still remains unknown.^9^, ^28^, ^29^ Here, we reported for the first that tectorigenin exerts anti‐osteoporosis effects in vivo and inhibits RANKL‐induced osteoclastogenesis in vitro. Thus, our study suggests that tectorigenin has a therapeutic potential for osteoporosis.

## 5 CONCLUSION

We demonstrate that tectorigenin inhibits RANKL‐induced osteoclastogenesis via suppression of NF‐κB signalling in vitro and could also reduce bone resorption in ovariectomized C57BL/6. These findings suggest that tectorigenin is a potential pharmacological choice for osteoporosis.

## CONFLICT OF INTEREST

No conflicts of interest have been reported by the authors or by any individuals in control of the content of this article.
