# Supplementary figures and images for: Tectorigenin inhibits RANKL‐induced osteoclastogenesis via suppression of NF‐κB signalling and decreases bone loss in ovariectomized C57BL/6
Source: J Cell Mol Med. 2018 Jul 31;22(10):5121–31. doi: 10.1111/jcmm.13801 (PMC6156464; doi:10.1111/jcmm.13801)

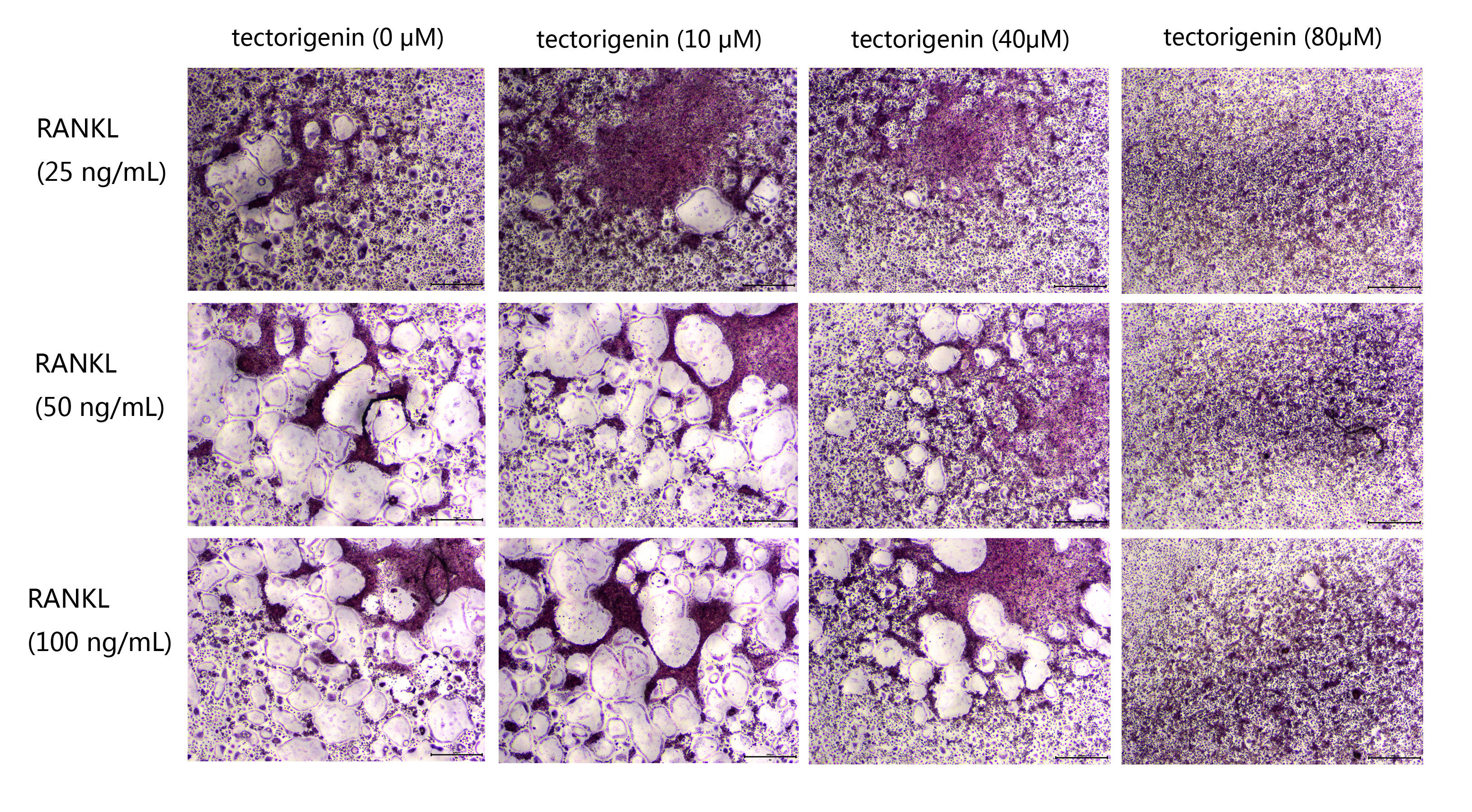

Supplement: Supplementary file 1 [file JCMM-22-5121-s001.tif]
